# The Relationship Between Mental Toughness, Job Loss, and Mental Health Issues During the COVID-19 Pandemic

**DOI:** 10.3389/fpsyt.2020.607246

**Published:** 2021-02-03

**Authors:** Dara Mojtahedi, Neil Dagnall, Andrew Denovan, Peter Clough, Sophie Hull, Derry Canning, Caroline Lilley, Kostas A. Papageorgiou

**Affiliations:** ^1^Department of Psychology, Centre for Cognition and Neuroscience, University of Huddersfield, Huddersfield, United Kingdom; ^2^Faculty of Health, Psychology and Social Care, Manchester Metropolitan University, Manchester, United Kingdom; ^3^School of Psychology, Queen's University Belfast, Belfast, United Kingdom

**Keywords:** COVID-19, mental health, mental toughness, unemployment (effects of), anxiety, depression, stress

## Abstract

Concerns toward public well-being and mental health are increasing considering the COVID-19 pandemic's global societal and individual impact. The present study builds on the current body of COVID-19 literature by examining the role of mental toughness (MT) in predicting negative affective states (depression, anxiety and stress) during the pandemic. The study also examined the effects of changes in employment on mental health and MT. Participants (*N* = 723) completed a battery of questionnaires including the *Mental Toughness Questionnaire 48-item, The State-Trait Anxiety Inventory*, and the *Depression, Anxiety and Stress Scale – 21 items*. Participants reported relatively higher levels of depression, stress and anxiety in comparison to pre-COVID-19 samples from previous research, with respondents who had lost their jobs during the pandemic reporting higher levels of negative affective states. Despite this, mentally tough individuals appeared to report lower levels of depression, anxiety and stress. Moreover, moderation analyses identified some interaction between MT and employment status when predicting depression, anxiety and stress. Our findings suggest that MT may have some utility in reducing the adverse mental health effects of the pandemic on individuals, however, further longitudinal research is needed to support these implications.

## Introduction

The COVID-19 pandemic has heightened concerns about public well-being and mental health (1). Correspondingly, there has been a rapid growth in research assessing the consequences of the coronavirus on psychological well-being. Since the effects are complex, evolving and ongoing, continuous research is required to explore the extent of the problem, and to identify potential protective factors. Acknowledging these points, the present study examined whether level of mental toughness (MT) predicted mental health outcomes during the pandemic and assessed whether high levels of MT moderated (reduced) the potential negative psychological effects of the COVID-19 pandemic. This included consideration of the consequences of occupational instability (i.e., job insecurity and loss), which research has identified as a major source of both social and individual concern [e.g., (2)].

Rajkumar (3), undertook a review of extant literature on COVID-19 and mental health. This revealed that symptoms of anxiety and depression (16–28%) and self-reported stress (8%) were common psychological reactions to the pandemic. Studies within the review noted also that stress and anxiety were frequently attendant with disturbed sleep quality (4, 5). Rajkumar (3) observed also that individual (e.g., mental health and age) and structural variables (e.g., support services) mediated and moderated risk. Pre-COVID literature identify further risk factors (e.g., negative affective temperaments & pre-existing depression) that could aggravate the negative psychiatric states experienced [e.g., feelings of hopelessness and increased suicide risk, (6, 7)]. Illustratively, due to the stress associated with the COVID-19 outbreak, patients with pre-existing mental disorders were susceptible to relapse or new episodes resulting from their disorder (8).

The general finding that self-reported anxiety, depression, and stress are common psychological reactions to the COVID-19 pandemic aligns with previous related work that has observed that psychological distress and symptoms of mental illness are associated with outbreaks of infectious disease (9, 10). In the context of COVID-19, resultant social and behavioral changes such as disrupted travel plans, social isolation, media information overload, and widespread panic buying of necessity goods, heightened the increasing menace of the epidemic. Collectively, these factors contributed to concern regarding the COVID-19 situation and helped to create a global atmosphere of concern and despair (9, 11).

An important feature of Rajkumar (3) review of COVID-19 and mental health was that it noted that individual and structural variables influenced the risk of negatives psychological consequences. In this context, a key factor is occupational security (2). Indeed, studies have noted that loss of employment and fear of unemployment are major concerns that contribute to negative affective states during the pandemic (12, 13). Relatedly, for many the pandemic has resulted in permanent or temporary (furlough) job loss, which have been previously linked with symptoms of depression (14). Despite global government attempts to relieve financial distress through increasing welfare support, Mimoun et al. (2) found that even those who were temporarily furloughed during the COVID-19 pandemic reported higher levels of distress than those who were unemployed prior to the pandemic. The authors explained that jobs “provide individuals a sense of confidence, self-esteem, and control” [(2, p. 184)].

Consideration of COVID-19-related literature supports the notion of individual differences in susceptibly to the pandemic's mental health impact. Wang et al. (15) surveyed the general public in China to better understand psychological impact (i.e., anxiety, depression, and stress) during the initial outbreak. They found that gender (i.e., female), student status, specific physical symptoms (e.g., myalgia, dizziness, coryza), and poor self-rated health status were significantly associated with greater psychological impact [i.e., higher levels of stress, anxiety, and depression as measured by the Depression Anxiety and Stress Scales, DASS21; (16)]. In a subsequent study, Wang et al. (17) conducted a longitudinal study covering the initial outbreak (Jan 31) and the peak of the epidemic 4 weeks later. During the preliminary evaluation, moderate-to-severe stress (8.1%), anxiety (28.8%) and depression (16.5%) were experienced by a noticeable minority of the group. Additionally, while the number of confirmed cases of COVID-19 increased markedly from the first to second survey, no significant changes occurred for DASS21 scores. Proposed protective factors included greater confidence in doctors, perceived survival likelihood and low risk of contracting COVID-19, satisfaction with health information, and personal precautionary measures.

From these studies it is clear that individual differences can play a significant role in mitigating the negative mental effects of the pandemic (15, 17, 18). Although adversity and challenge are natural consequences of everyday existence, susceptibility to the adverse consequences of accompanying anxiety, depression and stress can prove detrimental to mental health, well-being and everyday functioning (i.e., social, educational, occupational functioning & suicide ideation) (6, 19). Thus, with regards to the COVID-19 pandemic, it is important to identify and understand psychological factors that protect against potential commensurate anxiety, depression and stress. One widely researched positive psychological construct that has been associated with beneficial outcomes across a range of settings (e.g., educational, occupational and sport) is mental toughness (MT).

The concept of MT is highly relevant to the COVID-19 pandemic because it provides a conceptual framework for understanding individual differences in resilience and reactivity to negative impacts. At a general level, MT serves as an umbrella term to denote enabling psychological resources across a range of achievement contexts that promote positive mental health (20–22). The concept was initially employed within the domain of sport psychology to denote a battery of experientially developed and heritable psychological resources (i.e., values, attitudes, emotions, cognitions, and behaviors) that facilitated success in sports and physical activity (23). However, since its emergence, MT has been employed within clinical, developmental and occupational contexts, demonstrating similar enabling effects on achievement and positive mental health (24–32).

There are various conceptualisations of MT [e.g., (33, 34)]. The most widely cited and generally applied model was proposed by Clough and colleagues. Clough et al. (35) characterized MT as a composite of four interrelated, but independent components: [1] *Control* (life and emotion): the tendency to feel and act as if one is influential and keep anxieties in check; [2] *Commitment*: the tendency to be deeply involved in pursuing goals despite difficulties that arise; [3] *Challenge*: the tendency to see potential threats as opportunities for self-development and to continue to strive in changing environments; and [4] *Confidence* (in abilities and interpersonal): the belief that one is a truly worthwhile person in spite of setbacks, and the ability to push oneself forward in social settings.

Commensurate with previous work, the authors postulated that high levels of MT would attenuate the adverse psychological effects of the COVID-19 pandemic. This rationale derived from innumerable studies evidencing that individuals with higher levels of MT adapt better to stressful situations (36–38). For example, in a longitudinal study, Gerber et al. (37) explored the relationships between MT, psychological stress, depressive symptoms, and life satisfaction. Both perceived stress and depressive symptoms correlated negatively with MT. Moreover, MT was positively associated with life satisfaction. The researchers also found that well-adjusted individuals (low levels of stress, few depressive symptoms, and high life satisfaction) scored high on MT, whereas maladjusted individuals (high levels of stress, depressive symptoms, and little life satisfaction) tended to have lower levels of MT. Interestingly, resilient (moderate levels of stress at baseline, decreased depressive symptoms and increased life satisfaction at follow-up) and deteriorated (increasing levels of stress, increasing depressive symptoms, and decreasing life satisfaction) individuals did not differ at baseline but showed an increase/decline of MT over time (resilient and deteriorating individuals, respectively).

Consistent with these findings, Gerber et al. (37) showed that MT was associated with lower perceived stress and fewer depressive symptoms in a sample of 284 high school students and in a sample of 140 undergraduate students. They also showed that MT moderates the relationship between high perceived stress and depressive symptoms. More specifically, high levels of MT were associated with lower depressive symptoms, when perceived stress levels were high.

### Present Study

Research on the mental health implications of the COVID-19 pandemic is rapidly growing. However, relatively little academic work has attempted to identify dispositional protective factors against negative affective state during the pandemic (39). Previous research has identified a clear link between MT and resilience to stress, however, prior to this paper the relationship had not be explored in the context of the COVID-19 pandemic – a period of global and societal distress. Accordingly, the present study examined the relationship between MT and self-reported levels of depression, anxiety, and stress. Past research suggests that mentally tough individuals are less prone to experiencing negative emotions when placed in stressful situations (37, 38). Emanating from this, the current authors contend that mentally tough individuals should therefore be less susceptible to negative affective states during lockdown. More specifically, three hypotheses have been formulated: Hypothesis one predicts that MT traits will be negatively correlated with depression (DASS21); Hypothesis two predicts that MT traits will be negatively correlated with anxiety (DASS21 & STAI-Y1); and Hypothesis three predicts that MT traits will be negatively correlated with stress (DASS21).

Clough et al. (35) define MT as a stable, narrow, personality trait. This supposition is supported by consistent evidence of a genetic underpinning [e.g., (40–42)]. However, they recognize also that MT is modified by environmental factors, [e.g., training, (43); and positive youth experiences (44)]. Thus, it is possible that sustained and pervasive stressors may affect toughness scores. One objective stressor is job loss. Losing one's job often has a negative effect on well-being (14). Uniquely, in the current pandemic there are four options: retaining the job, furlough, job loss and previously unemployed. Thus, the second aim of the study was to examine whether employment status had an effect on MT scores and negative affective state. Due to a lack of research exploring the stability of MT, no predictions were made on the relationship between employment status and MT. However, the effects of job loss on mental health have been observed within past research [see (2, 14)]. Based on these findings, the following additional hypotheses are presented: Hypothesis four predicts that respondents who had lost their jobs during the pandemic will report higher level depression (DASS21) than those in employment; Hypothesis five predicts that respondents who had lost their jobs during the pandemic will report higher level anxiety (DASS21 & STAI-Y1) than those in employment; and Hypothesis six predicts that respondents who had lost their jobs during the pandemic will report higher levels of stress (DASS21) than those in employment.

The present study is the first to examine the role of MT during the COVID-19 pandemic and does so using a large diverse sample (internationally and temporally, see below) to produce representative results. The research will allow us to determine whether such traits can mitigate mental health problems during the pandemic. Moreover, the practical implications of the findings can inform future strategies for protecting public mental health during current and future pandemics.

## Materials and Methods

### Samples and Design

This study was cross-sectional in nature. The cross-sectional approach is frequently criticized because it is inclined to common method variance (CMV) (45). This occurs when variations in responses reflect measurement procedure rather than underlying differences in the observed construct(s). To counter CMV, the researchers employed procedural remedies (46). Firstly, instructions created psychological distance between scales by emphasizing that each measure assessed a separate construct. Encouraging respondents to perceive scales as distinct has previously successfully reduced common method variance (47). Secondly, the instructions attempted to negate social desirability effects and evaluation apprehension by stating that there were no correct answers. Published studies have previously successfully implemented these procedural remedies [e.g., (48, 49)].

The study used self-report measures hosted online via Qualtrics. Data collection occurred at two different time-points on independent samples to determine whether the association between MT and negative states could be replicated. The inclusion criteria required all participants to be aged 18 or above and speak English proficiently. The combined dataset consisted of 723 participants (male = 315, female = 407, and other = 1), aged between 18 and 78 (*M* = 35.06, *SD* = 13.65). The demographic details of both samples are presented in Table 1. The first sample (Sample A) consisted of 376 participants (male = 95, female = 280, other = 1) aged between 18 and 78 (*M* = 34.10, *SD* = 14.34) from the UK and Ireland. Data collection was carried out between April 23rd and May 21st, with most responses (76.33%) collected between April 23rd and April 30th, 2020. The survey was advertised through social media and online internet groups. Participants from Sample A were not financially compensated for their involvement.

**Table 1:** Demographic variables for samples.

	**Sample A**	**Sample B**	**Total**
	**(*n* = 376)**	**(*n* = 347)**	**(*N* = 723)**
Age (*M, sd)*	34.1 (14.34)	36.09 (12.79)	35.06 (13.65)
Gender			
Male	95 (25.3%)	220 (63.4%)	315 (43.6%)
Female	280 (74.5%)	127 (36.6%)	407 (56.3%)
Other	1 (0.3%)	0	1 (0.1%)
Employment			
Job loss	14 (3.9%)	50 (14.4%)	64 (9.1%)
Furloughed	75 (21%)	31 (8.9%)	106 (15.1%)
Previously unemployed	63 (17.6)	46 (13.3%)	109 (15.5%)
Working (traveling)	80 (22.4%)	66 (19%)	146 (20.7%)
Working (home)	125 (35%)	154 (44%)	279 (36.9%)

Data for the second sample (Sample B) were collected on May 18th and May 25th, 2020, with the majority (97.4%) of responses collected on May 25th. For Sample B, the authors recruited participants via an online crowd sourcing marketplace, Amazon Mechanical Turk (*Mturk*). Each respondent was rewarded $0.30 for their involvement. Previous research indicates that data collected through *Mturk* are of high quality (50). Additionally, measures were taken to ensure that respondents were reading and responding to the questions logically (as opposed to haphazardly providing responses to receive the reward).

Three validity-test questions were placed within the survey instructing participants to select a specific response (e.g., “for this question please select the number 4”). In total 415 participants were recruited. However, 68 cases were omitted from the study after failing to correctly answer the validity-test questions, leaving a final sample of 347 participants (male = 220, female = 127) aged between 18 and 76 (*M* = 36.09, *SD* = 12.79). In order to allow for cultural comparisons to be made between the two samples, the authors used *Mturk's* preference filter to make the Sample B survey only available to non-UK participants, however, 17 participants from the UK still managed to complete the survey and were included in the final sample. The other participants from Sample B were from North America (*n* =239), India (*n* = 60), and Brazil (*n* = 18) and other (*n* = 13).

The survey asked questions pertaining to the participants' (i) demographic information (including job status), (ii) negative affective states during the pandemic (i.e., anxiety, depression, and stress), and (iii) mental toughness. The survey also contained some additional questions about the participants' general well-being and attitudes toward COVID-19, however these items were not of interest to the present study and thus, they are not discussed further. The average completion time was 21 min for respondents in Sample A and 15 min for respondents in Sample B.

### Measures

Respondents provided information about their age, gender, country of residence and job status during the pandemic. For job status, participants were asked to select the most appropriate response from the following options: *Unemployed before the pandemic*, I *lost my job/business during the pandemic, furloughed, I still have my job/business and travel to work, I still have my job/business and working from home (WFH)*.

*Depression, Anxiety, and Stress Scale* [DASS21; (51)] is a 21-item self-report instrument that measures symptoms of depression, anxiety and stress at the time of participation. Each item is presented as a statement that the participant rates their agreement to using a four-point Likert scale (0 = Did not apply to me at all, 3 = Applied to me very much or most of the time). The scores for each subscale are calculated by multiplying the sum of the respective items by two. DASS21 was identified as a suitable measure for the present study due to demonstrating high internal consistency across clinical and non-clinical samples (52–54). High Cronbach's alphas were observed within the present study for Sample A (Stress = 0.9, Anxiety = 0.82, Depression = 0.91) and B (Stress = 0.91, Anxiety = 0.92, and Depression = 0.92).

*Spielberger State-Trait Anxiety Inventory* [STAI; (55)] measures trait (baseline) and state (situational) anxiety, through two 20-item scales. The items describe different affective states and participants are required to indicate how much each statement reflects their mood either at the time of survey completion (STAI-Y1) or in general (STAI-Y2) using a four-point Likert scale (1 = almost never/not at all, 4 = almost always/very much so). Research studies [e.g., (56, 57)] have continuously supported the construct validity of both subscales. High Cronbach's alphas were also observed within the present study within Sample A (STAI-Y1 = 0.96; STAI-Y2 = 0.94) and B (STAI-Y1 = 0.91; STAI-Y2 = 0.92).

The *Mental Toughness Questionnaire 48* [MTQ48; (35)] measures MT through four components: Control (14 items), Confidence (15 items), Commitment (11 items) and Challenge (8 items). Participants are required to indicate their level of agreement using a five-point Likert scale (1 = strongly disagree; 5 = strongly agree). Each component is scored by calculating the mean of the respective items with higher scores indicating a greater level of MT. The MTQ-48 has established internal and test–retest reliability (36, 37, 40, 58, 59). Furthermore, Clough et al. (35) provide evidence for MTQ-48 construct validity via significant relationships with related measures (i.e., optimism, self-image, satisfaction with life, self-efficacy, and trait anxiety). High Cronbach's alphas were also observed within the present study within Sample A (Challenge = 0.82; Commitment = 0.86; Control = 0.77; and Confidence = 0.88) and B (Challenge = 0.67; Commitment = 0.79; Control = 0.68; and Confidence = 0.78).

### Statistical Analysis

All statistical analyses were performed using SPSS® 26.0 (IBM Corporation, Armonk NY, USA) for Windows®/Apple Mac®. For all regression models, preliminary analyses were conducted to ensure no violation of the assumptions of linearity, and homoscedasticity. The collinearity statistics (VIF & Tolerance) for all models indicated that multicollinearity was unlikely to be a problem [see (60)]. All predictor variables were statistically correlated with the outcome variables which indicates that the data was suitably correlated with the dependent variables for examination through multiple linear regression to be reliably undertaken. All measures of effect size were interpreted in accordance with Cohen (61).

## Results

### Depression, Anxiety, and Stress During the COVID-19 Pandemic

Preliminary observations were conducted to test the normality assumptions of the dependent variables. Observations of the histograms indicated that the DASS21 variables (depression, anxiety, and stress) were not normally distributed. As a result, between-group comparisons of DASS21 scores were conducted using non-parametric tests. Following this, a series of *t*-tests and Mann-Whitney *U*-tests were conducted to compare the two samples in mental toughness and affective states (see Table 2). Bonferroni corrections were applied (corrected to *p* = 005).

**Table 2:** Scale averages.

	**Sample A (*****n*** **=** **372)**		**Sample B (*****n*** **=** **347)**		**Statistics**
	***m***	***sd***	***m***	***sd***	
State anxiety	43.58	12.89	45.55	10.86	*p = 0.03* [*t*(686.67) = −2.2, 95% CI = −3.74 to −0.21, η^2^ = 0.007]^t^
Trait anxiety	42.7	12.19	44.36	10.95	*p = 0.0*6 [*t*(702.79)= −1.92, 95% CI= −3.38 to 0.04, η^2^ = 0.005]^t^
Stress	13.32	10.05	17.48	11.02	*p* > 0.001 [*U* = 49,741.5, *Z* = −5.22, *r* = −0.19]^m^
Anxiety	7.01	7.78	15.20	11.71	*p > 0.001 [U = 39,863, Z =* –*8.9, r =* –*0.33]^m^*
Depression	10.99	9.9	16.32	11.35	*p* > 0.001 [*U* = 47,354.5, *Z* = −6.14, *r* = −0.23]^m^
Challenge	3.47	0.62	3.41	0.54	*p* = 0.18 [*t*(713.98) = 1.35, 95% CI = −0.03 to 0.14, η^2^ = 0.002]^t^
Commitment	3.46	0.64	3.32	0.61	*p* = 0.002 [*t*(717) = 3.13, 95% CI = 0.05 to 0.24, η^2^ = 0.01]^t^
Control	3.14	0.53	3.08	0.45	*p* = 0.39 [*t*(714.09) = 0.86, 95% CI = −0.04 to 0.1, η^2^ = 0.001]^t^
Confidence	3.25	0.65	3.29	0.5	*p* = 0.35 [*t*(702.21) = −0.94, 95% CI = −0.13 to 0.04, η^2^ = 0.001]^t^

corrected alpha = 0.005;

t = t-test (two-tailed),

m *= Mann-Whitney U*.

There were no significant differences between the two samples in state anxiety and trait anxiety at the corrected alpha. In relation to the DASS21 variables (depression, anxiety, and stress), preliminary observations indicated that the data was not normally distributed, therefore Mann-Whitney U tests were used to compare the samples. For stress, 27.5% of Sample A reported moderate to extremely severe levels of stress compared to 47.8% of Sample B [see (51) for label scoring]. Differences in stress scores were found to be statistically significant, but small. For anxiety, 31% of Sample A reported moderate to extremely severe levels of anxiety compared to 63.7% of Sample B. Differences in anxiety scores were also significant, but small. For depression, 33.1% of Sample A reported moderate to extremely severe levels of depression compared to 62.6% of Sample B. Differences in depression scores were also significant, but small. In relation to MT, differences in Commitment reached statistical significance, however, the eta squared statistic indicated a small effect size (see Table 2). No significant differences were found for Challenge, Control, and Confidence.

### Mental Toughness and Negative Affective States

A series of hierarchical multiple regressions (HMR) were performed to investigate the ability of MT traits (Challenge, Commitment, Control, & Confidence) to predict depression, anxiety and stress. To reduce the effects of individual differences in baseline negative affectivity, a hierarchical model was used to control for trait anxiety (STAI-Y2). In the first step of HMR, four predictors were entered: Challenge, Commitment, Control, & Confidence; the second step then introduced trait anxiety to the model. Due to the sample comparisons identifying significant differences in depression, anxiety and stress, the association between MT traits and negative affective states were assessed for each sample individually. The correlations between all continuous variables are presented in Table 3. The table suggests that the correlations between the variables were relatively similar across both samples. The correlations between MT traits and negative affective states were small to large (*r* = −0.31 to −0.77). All predictor variables were statistically correlated with depression, anxiety (DASS21 and STAI-Y1) and stress, which indicates that the data was suitably correlated with the dependent variables for examination through multiple linear regression. The HMR model properties for Sample A and B are presented in Tables 4, 5, respectively.

**Table 3:** Correlations between continuous variables.

	**Sample A**									**Sample B**								
	**1**	**2**	**3**	**4**	**5**	**6**	**7**	**8**	**9**	**1**	**2**	**3**	**4**	**5**	**6**	**7**	**8**	**9**
1. State anxiety	1	0.74	0.79	0.66	0.72	−0.49	−0.43	−0.61	−0.57	1	0.77	0.6	0.47	0.6	−0.52	−0.57	−0.65	−0.63
2.Trait anxiety		1	0.64	0.63	0.71	−0.6	−0.62	−0.74	−0.77		1	0.75	0.64	0.77	−0.57	−0.73	−0.77	−0.74
3. Stress (dass)			1	0.72	0.72	−0.4	−0.4	−0.55	−0.48			1	0.87	0.89	−0.31	−0.63	−0.62	−0.49
4. Anxiety (Dass)				1	0.68	−0.44	−0.42	−0.56	−0.51				1	0.85	−0.25	−0.59	−0.57	−0.4
5 Depression (Dass)					1	−0.46	−0.51	−0.57	−0.61					1	−0.33	−0.69	−0.67	−0.54
6.Challenge (MT)						1	0.63	0.67	0.7						1	0.61	0.61	0.72
7.Commitment (MT)							1	0.66	0.67							1	0.76	0.74
8.Control (MT)								1	0.77								1	0.76
9.Confidence (MT)									1									1

*All relationships were statistically significant (p < 0.001)*.

**Table 4:** Hierarchical multiple regression models for Sample A (*n* =372).

	**Stress**								**Depression**								**State anxiety (STAI-Y1)**								**Anxiety (DASS21)**							
	***R***	***R^**2**^***	***R^**2**^ Change***	***B***	***SE***	**β**	***t***		***R***	***R^**2**^***	***R^**2**^ Change***	***B***	***SE***	**β**	***t***		***R***	***R^**2**^***	***R^**2**^ Change***	***B***	***SE***	**β**	***t***		***R***	***R^**2**^***	***R^**2**^ Change***	***B***	***SE***	**β**	***t***
Step 1	0.56	0.31^***^							0.64	0.41^***^							0.63	0.4^***^							0.57	0.33^***^					
Challenge				−0.16	1.09	−0.01	−0.15					0.72	1	0.05	0.72					−1.73	1.33	−0.08	−1.3					−0.76	0.83	−0.06	−0.92
Commitment				−0.38	1.01	−0.02	−0.37					−2.25	0.92	−0.15^*^	−2.43					0.97	1.23	0.05	0.79					−0.38	0.77	−0.03	−0.49
Control				−8.05	1.42	−0.43^***^	−5.67					−3.69	1.3	−0.2^**^	−2.85					−10.11	1.73	−0.42^***^	−5.85					−5.35	1.08	−0.37^***^	−4.94
Confidence				−2.04	1.22	−0.13	−1.68					−6.03	1.11	−0.39^***^	−5.42					−4.36	1.48	−0.22^**^	−2.94					−2.01	0.93	−0.17^*^	−2.17
Step 2	0.66	0.43^***^	0.12^***^						0.72	0.52^***^	0.11^***^						0.76	0.57^***^	0.17^***^						0.65	0.42^***^	0.09^***^				
Challenge				−0.21	1	−0.01	−0.21					0.68	0.9	0.04	0.75					−1.8	1.13	−0.09	−1.6					−0.8	0.78	−0.06	−1.02
Commitment				0.55	0.93	0.04	0.59					−1.38	0.84	−0.09	−1.63					2.4	1.05	0.12^*^	2.29					0.2*5*	0.73	0.02	0.34
Control				−4.4	1.36	−0.23^**^	−3.23					−0.26	1.24	−0.01	−0.21					−4.45	1.54	−0.18^**^	−2.89					−2.89	1.06	−0.2^**^	−2.72
Confidence				1.92	1.2	0.12	1.6					−2.29	1.09	−0.15^*^	−2.1					1.79	1.36	0.09	1.32					0.66	0.94	0.06	0.71
Trait anxiety				0.48	0.06	0.58^***^	8.5					0.45	0.05	0.55^***^	8.83					0.74	0.06	0.7^***^	11.66					0.32	0.04	0.5^***^	7.33

Statistical significance:

* p < 0.05;

** p < 0.01;

*** *p < 0.001*.

**Table 5:** Hierarchical multiple regression models for Sample B (*n* = 347).

	**Stress**								**Depression**								**State anxiety (STAI–Y1)**								**Anxiety (DASS21)**							
	***R***	***R^**2**^***	***R^**2**^ Change***	***B***	***SE***	**β**	***t***		***R***	***R^**2**^***	***R^**2**^ change***	***B***	***SE***	**β**	***t***		***R***	***R^**2**^***	***R^**2**^ change***	***B***	***SE***	**β**	***t***		***R***	***R^**2**^***	***R^**2**^ change***	***B***	***SE***	**β**	***t***
Step 1	0.69	0.47^***^							0.74	0.55^***^							0.68	0.47^***^							0.65	0.43^***^					
Challenge				4.45	1.17	0.22^***^	3.81					5.14	1.11	0.24^***^	4.64					−1.85	1.16	−0.09	−1.6					4.56	1.29	0.21^***^	3.53
Commitment				−7.93	1.18	−0.44^***^	−6.73					−9.05	1.12	−0.49^***^	−8.1					−1.18	1.17	−0.07	−1.01					−9.71	1.3	−0.51^***^	−7.44
Control				−9.84	1.61	−0.42^***^	−6.12					−10.03	1.53	−0.41^***^	−6.57					−8.48	1.59	−0.36^***^	−5.32					−10.38	1.78	−0.41^***^	−5.82
Confidence				−0.1	1.52	−0.004	−0.06					−0.92	1.45	−0.04	−0.63					−4.89	1.51	−0.24^**^	−3.24					3.15	1.69	0.14	1.87
Step 2	0.79	0.63^***^	0.16^***^						0.82	0.68^***^	0.13^***^						0.78	0.61^***^	0.14^***^						0.72	0.52^***^	0.09^***^				
Challenge				4.39	0.98	0.22^***^	4.46					5.08	0.94	0.24^***^	5.4					−1.9	1.	−0.1	−1.91					4.51	1.19	0.21^***^	3.79
Commitment				−5.11	1.02	−0.29^***^	−5.02					−6.43	0.98	−0.35^***^	−6.6					1.42	1.04	0.08	1.37					−7.42	1.23	−0.39^***^	−6.02
Control				−3.84	1.45	−0.16^**^	−2.65					−4.46	1.38	−0.18^**^	−3.22					−2.95	1.47	−0.13^*^	−2.01					−5.51	1.75	−0.22^**^	−3.15
Confidence				4.09	1.33	0.19^**^	3.08					2.98	1.27	0.14^*^	2.34					−1.03	1.35	−0.05	−0.76					6.55	1.61	0.29^***^	4.07
Trait anxiety				0.68	0.06	0.68^***^	11.89					0.64	0.06	0.61^***^	11.56					0.63	0.06	0.64^***^	10.78					0.56	0.07	0.52^***^	7.99

Statistical significance:

* p < 0.05;

** p < 0.01;

*** *p < 0.001*.

### Stress

A HMR model was used to predict stress within Sample A. In the first step of hierarchical multiple regression, the model was statistically significant *F*_(4,350)_ = 39.6; *p* < 0.001 and explained 31.2% of variance in state stress. Control made a significant unique contribution to the model (see Table 4). After entry of trait anxiety at Step 2 the total variance explained by the model as a whole was 42.1% [*F*_(5,349)_ = 52.57; *p* < 0.001]. The introduction of trait anxiety explained an additional 11.8% of variance in state stress, after controlling for the mental toughness traits [*F*_(1,349)_ = 72.2; *p* < 0.001]. In the final adjusted model, trait anxiety and Control were statistically significant.

For Sample B, in the first step, the model was statistically significant *F*_(4,342)_ = 76.53; *p* < 0.001 and explained 47.2% of variance in state stress. Three predictors made a significant unique contribution to the model (see Table 5). After entry of trait anxiety at Step 2 the total variance explained by the model as a whole was 62.7% [*F*_(5,341)_ = 114.64; *p* < 0.001]. The introduction of trait anxiety explained an additional 15.5% of variance in state stress, after controlling for the mental toughness traits [*F*_(1,341)_ = 141.4; *p* < 0.001]. In the final adjusted model, all five predictor variables were statistically significant.

### Depression

A HMR was next used to predict depression within Sample A. In the first step, the model was statistically significant *F*_(4,351)_ = 60.53; *p* < 0.001 and explained 41% of variance in state depression. Three of the four mental toughness traits made a significant unique contribution to the model (see Table 4). After entry of trait anxiety at Step 2 the total variance explained by the model as a whole was 52% [*F*_(5,350)_ = 60.46; *p* < 0.001]. The introduction of trait anxiety explained an additional 11% of variance in state depression, after controlling for the mental toughness traits [*F*_(1,350)_ = 77.89; *p* < 0.001]. In the final adjusted model, trait anxiety and Confidence were statistically significant.

For Sample B, in the first step, the model was statistically significant *F*_(4,342)_ = 105.41; *p* < 0.001 and explained 55.2% of variance in state depression. Three of the four mental toughness traits made a significant unique contribution to the model (see Table 5). After entry of trait anxiety at Step 2 the total variance explained by the model as a whole was 67.8% [*F*_(5,341)_ = 143.71; *p* < 0.001]. The introduction of trait anxiety explained an additional 12.6% of variance in state depression, after controlling for the mental toughness traits [*F*_(1,341)_ = 133.52; *p* < 0.001]. In the final adjusted model, all five predictor variables were statistically significant.

### State Anxiety (STAI-Y1)

A HMR was next used to predict state anxiety (STAI-Y1) within Sample A. In the first step, the model was statistically significant *F*_(4,339)_ = 56.06; *p* < 0.001 and explained 40% of variance in state anxiety. Two of the four mental toughness traits made a significant unique contribution to the model (see Table 4). After entry of trait anxiety at Step 2 the total variance explained by the model as a whole was 57% [*F*_(5,338)_ = 89.89; *p* < 0.001]. The introduction of trait anxiety explained an additional 17.3% of variance in state anxiety, after controlling for the mental toughness traits [*F*_(1,338)_ = 135.96; *p* < 0.001]. In the final adjusted model, trait anxiety, Control and Commitment were statistically significant.

For Sample B, in the first step, the model was statistically significant *F*_(4,342)_ = 75.12; *p* < 0.001 and explained 46.8% of variance in state anxiety. Two of the four mental toughness traits made a significant unique contribution to the model (see Table 5). After entry of trait anxiety at Step 2 the total variance explained by the model as a whole was 60.3% [*F*_(5,341)_ = 103.58; *p* < 0.001]. The introduction of trait anxiety explained an additional 13.5% of variance in state anxiety, after controlling for the mental toughness traits [*F*_(1,341)_ = 116.21; *p* < 0.001]. In the final adjusted model, trait anxiety and Control were statistically significant.

### Anxiety (DASS21)

A HMR was next used to predict anxiety (DASS21) within Sample A. In the first step, the model was statistically significant *F*_(4,351)_ = 43.04; *p* < 0.001 and explained 33% of variance in anxiety (DASS21). Two of the four mental toughness traits made a significant unique contribution to the model (see Table 4). After entry of trait anxiety at Step 2 the total variance explained by the model as a whole was 42% [*F*_(5,338)_ = 89.89; *p* < 0.001]. The introduction of trait anxiety explained an additional 8.9% of variance in anxiety (DASS21), after controlling for the mental toughness traits [*F*_(1,350)_ = 53.78; *p* < 0.001]. In the final adjusted model, trait anxiety and Control were statistically significant.

For Sample B, in the first step, the model was statistically significant *F*_(4,342)_ = 63.76; *p* < 0.001 and explained 42.7% of variance in anxiety (DASS21). Three of the four mental toughness traits made a significant unique contribution to the model (see Table 5). After entry of trait anxiety at Step 2 the total variance explained by the model as a whole was 51.7% [*F*_(5,341)_ = 73.14; *p* < 0.001]. The introduction of trait anxiety explained an additional 9% of variance in anxiety (DASS21), after controlling for the mental toughness traits [*F*_(1,341)_ = 63.82; *p* < 0.001]. In the final adjusted model, all five predictor variables were statistically significant.

### Employment Status as a Predictor of Mental Toughness and Affective State

As illustrated in the Table 1, there were some proportional differences in employment status between the samples. Most notably, very few participants from Sample A had lost their job/business during the pandemic, in comparison to Sample B. To ensure that all employment status groups had a sufficient number of cases for reliable comparisons to be made, data from the two samples were combined for the subsequent analyses.

### Employment Status and Mental Toughness

A between-groups multivariate analysis of variance was conducted to explore the impact of employment status on MT traits (Challenge, Commitment, Control, & Confidence). Descriptive and inferential statistics for MT differences between employment status groups are presented in Table 6. There was a statistically significant main effect for employment, [*F*_(16, 2123.9)_ = 2.56, *p* = 0.001; Wilks' Λ = 0.94]. Univariate comparisons found a significant main effect for job outcome on all MT traits, inferential statistics are presented in Table 6. Further *post-hoc* Tukey comparisons are presented in Table 7 and discussed below.

**Table 6:** MT scores for job outcome group.

	**Job outcome**					
	**Job loss**	**Furloughed**	**Previously unemployed**	**Working (traveling)**	**Working (home)**	**Statistics**
*N*	64	106	109	146	279	
	M (SD)	M (SD)	M (SD)	M(SD)	M (SD)	
Challenge	3.3 (0.55)	3.31 (0.54)	3.51 (0.66)	3.42 (0.59)	3.49 (0.55)	*F*_(4,698)_ = 0.3.34, *p* = 0.01
Commitment	3.14 (0.5)	3.31 (0.62)	3.44 (0.74)	3.35 (0.62)	3.48 (0.61)	*F*_(4,698)_ = 4.66, *p* = 0.001
Control	2.89 (0.42)	3 (0.5)	3.22 (0.58)	3.08 (0.47)	3.2 (0.46)	*F*_(4,698)_ = 8.56, *p* < 0.001
Confidence	3.08 (0.52)	3.15 (0.6)	3.31 (0.73)	3.24 (0.56)	3.35 (0.53)	*F*_(4,698)_ = 4.54, *p* = 0.001

**Table 7:** Significant *post–hoc* comparisons for job outcome and MT.

	**Statistics**		
	***p***	**95% CI**	***d***
Challenge			
Furloughed < WFH	0.05	−0.36 to −0.001	0.33
Commitment			
Job loss < WFH	0.001	−0.57 to −0.1	0.57
Job loss < previously unemployed	0.02	−0.57 to −0.03	0.44
Control			
Job loss < WFH	< 0.001	−0.5 to −0.13	0.69
Job loss < previously unemployed	< 0.001	−0.54 to −0.13	0.63
Furloughed < WFH	0.004	−35 to −0.04	0.42
Furloughed < previously unemployed	0.01	−0.4 to −0.04	0.41
Confidence			
Job loss < WFH	0.01	0.05 to 0.5	0.51
Furloughed < WFH	0.02	0.02 to 0.39	0.36

*Post-hoc* comparisons for Challenge scores indicated that the mean score for furloughed participants was significantly lower than WFH participants; the difference between the groups was small. For Commitment scores, *post-hoc* tests indicated that the mean score for Commitment in job/business loss participants was significantly lower than WFH participants and previously unemployed participants; these differences were moderate and small, respectively. In relation to Control scores, *post-hoc* tests indicated that the mean score for job/business loss participants was significantly lower than WFH participants and previously unemployed participants. Both differences were moderate. Additionally, the mean score for Control in furloughed participants was significantly lower than WFH participants and previously unemployed participants. The differences here were small. Finally, for Confidence, *post-hoc* comparisons indicated that the mean score for WFH participants was significantly higher than furloughed participants and job/business loss participants. The differences were small and moderate, respectively.

### Employment Status and Negative Affective State

Lastly, four moderation analyses were carried out using hierarchical regressions to determine whether MT traits moderated the effects of different job outcomes on negative affective states (state anxiety, DASS21 anxiety, depression and stress). A dummy coding procedure was used to test the predictive abilities of each individual job outcome, with WFH as the reference category. The moderator variables (Challenge, Commitment, Control, & Confidence) were centered to allow the effect of the predictor to be distinguishable from the interaction. For each regression, the first step included the predictor (job outcome) and centered moderator variables (Challenge, Commitment, Control, & Confidence) and the second step introduced the interactions between these variables. The inferential properties for all four models are parented in Table 8.

**Table 8:** Hierarchical multiple regressions for emotional states.

	**SA**							**Depression**							**Anxiety**							**Stress**					
	***R***	***R^**2**^***	***B***	***SE***	**β**	***t***		***R***	***R^**2**^***	***B***	***SE***	**β**	***t***		***R***	***R^**2**^***	***B***	***SE***	**β**	***t***		***R***	***R^**2**^***	***B***	***SE***	**β**	***t***
Step 1	0.66	0.43						0.68	0.46						0.69	0.34						0.62	0.38				
Job loss			3.6	1.29	0.09^**^	2.79				4.49	1.14	0.12^***^	3.92				4.68	1.23	0.13^***^	3.81				3.59	1.2	0.1^**^	3.01
Furloughed			−0.02	1.05	−0.001	−0.02				−0.31	0.93	−0.01	−0.34				−0.88	1.	−0.03	−0.87				−1.33	0.97	−0.04	−1.36
Working (t)			0.6	0.94	0.02	0.64				0.1	0.83	0.004	0.12				1.81	0.89	0.07^*^	2.03				1.59	0.87	0.06	1.84
P.Unemployed			−0.65	1.03	−0.02	−0.63				−1.52	0.92	−0.05	−1.66				−1.63	0.99	−0.05	−1.65				−1.56	0.96	−0.05	−1.63
Challenge			−1.93	0.88	−0.09^*^	−2.2				2.75	0.78	0.15^***^	3.54				1.45	0.83	0.08	1.74				1.85	0.81	0.1^*^	2.28
Commitment			−0.22	0.82	−0.01	−0.27				−6.27	0.73	−0.36^***^	−8.59				−5.9	0.78	−0.35^***^	−7.52				−4.34	0.76	−0.26^***^	−5.7
Control			−9.15	1.17	−0.38^***^	−7.8				−6.58	1.04	−0.3^***^	−6.34				−7.83	1.12	−0.37^***^	−7.01				−9.12	1.09	−0.43^***^	−8.4
Confidence			−4.23	1.03	−0.21^***^	−4.12				−2.87	0.91	−0.15^**^	−3.15				1.75	0.98	0.1	1.79				−0.42	0.95	−0.02	−0.44
Step 2	0.67	0.45						0.69	0.47						0.61	0.37						0.64	0.41				
Job loss			4.23	1.49	0.1^**^	2.85				4.22	1.32	0.11^**^	3.2				4.89	1.4	0.13^**^	3.48				4.89	1.37	0.13^***^	3.57
Furloughed			−0.19	1.07	−0.01	−0.17				−0.02	0.95	−0.001	−0.03				−0.77	1.01	−0.03	−0.76				−1.21	0.99	−0.04	−1.23
Working (t)			0.71	0.94	0.02	0.75				0.012	0.83	0.0004	0.02				1.64	0.88	0.06	1.86				1.48	0.86	0.06	1.72
P.Unemployed			−0.29	1.05	−0.01	−0.27				−1.51	0.93	−0.05	−1.62				−1.7	0.99	−0.06	−1.71				−1.49	0.97	−0.05	−1.54
Challenge			−4.88	1.46	−0.24^**^	−3.36				1.82	1.29	0.1	1.41				2.15	1.38	0.12	1.57				2.74	1.34	0.15^*^	2.04
Commitment			−0.02	1.44	−0.001	−0.02				−5.36	1.28	−0.31^***^	−4.18				−5.74	1.36	−0.34^***^	−4.21				−4.63	1.33	−0.27^**^	−3.48
Control			−5.93	1.98	−0.25^**^	−3.				−7.4	1.75	−0.34^***^	−4.22				−9.29	1.87	−0.44^***^	−4.97				−10.32	1.82	−0.48^***^	−5.67
Confidence			−3.57	1.73	−0.18^*^	−2.07				−1.42	1.53	−0.08	−0.93				2.38	1.63	0.13	1.46				0.7	1.59	0.04	0.44
JLxChal			2.41	3.44	0.03	0.7				−1.18	3.05	−0.02	−0.39				−4.74	3.25	−0.07	−1.46				−6.42	3.17	−0.1^*^	−2.03
FxChal			3.19	2.77	0.06	1.15				3.2	2.46	0.06	1.3				−0.07	2.62	−0.001	−0.03				−0.16	2.55	−0.003	−0.06
WxChal			5.5	2.46	0.12^*^	2.24				1.72	2.19	0.04	0.78				−2.42	2.33	−0.06	−1.04				−0.65	2.27	−0.02	−0.29
PUxChal			4.6	2.47	0.1	1.84				−0.06	2.19	−0.001	−0.03				−1.02	2.34	−0.03	−0.44				−2.53	2.28	−0.06	−1.11
JLxComm			−1.7	3.03	−0.02	−0.55				−3.59	2.69	−0.05	−1.34				−5.36	2.87	−0.08	−1.87				−2.31	2.79	−0.04	−0.83
FxComm			4.12	2.55	0.08	1.62				1.11	2.26	0.02	0.49				3.57	2.41	0.08	1.48				1.37	2.34	0.03	0.59
WxComm			−3.23	2.34	−0.08	−1.38				−3.73	2.07	−0.1	−1.8				−3.88	2.21	−0.1	−1.76				−0.99	2.15	−0.03	−0.46
PUxComm			−0.01	2.37	0.0003	−0.01				0.39	2.11	0.01	0.18				3.31	2.24	0.09	1.48				3.6	2.19	0.1	1.65
JLxCont			−0.37	4.11	−0.004	−0.09				0.76	3.64	0.01	0.21				5.18	3.88	0.07	1.33				9.96	3.78	0.13^**^	2.63
FxCont			−8.9	3.62	−0.15^*^	−2.45				4.13	3.21	0.07	1.29				4.19	3.42	0.08	1.22				3.36	3.33	0.06	1.01
WxCont			−3.23	3.28	−0.06	−0.98				−2.33	2.91	−0.05	−0.8				−0.87	3.1	−0.02	−0.28				−3.64	3.02	−0.07	−1.21
PUxCont			−6.31	3.42	−0.12	−1.85				2.38	3.03	0.05	0.79				1.74	3.23	0.04	0.54				1.78	3.14	0.04	0.57
JLxConf			0.57	4.18	0.01	0.14				1.71	3.71	0.03	0.46				5.52	3.95	0.08	1.4				1.98	3.85	0.03	0.51
FxConf			−0.97	3.1	−0.02	−0.31				−6.91	2.75	−0.15^*^	−2.51				−5.6	2.93	−0.12	−1.91				−4.06	2.85	−0.09	−1.42
WxConf			0.37	3.1	0.01	0.12				0.93	2.75	0.02	0.34				4.1	2.93	0.1	1.4				2.79	2.86	0.07	0.98
PUxConf			−1.48	2.76	−0.04	−0.54				−2.98	2.45	−0.08	−1.21				−3.26	2.61	−0.09	−1.25				−4.31	2.55	−0.11	−1.69

* P < 0.05,

** P < 0.01,

*** *P < 0.005*.

**Figure 1:**
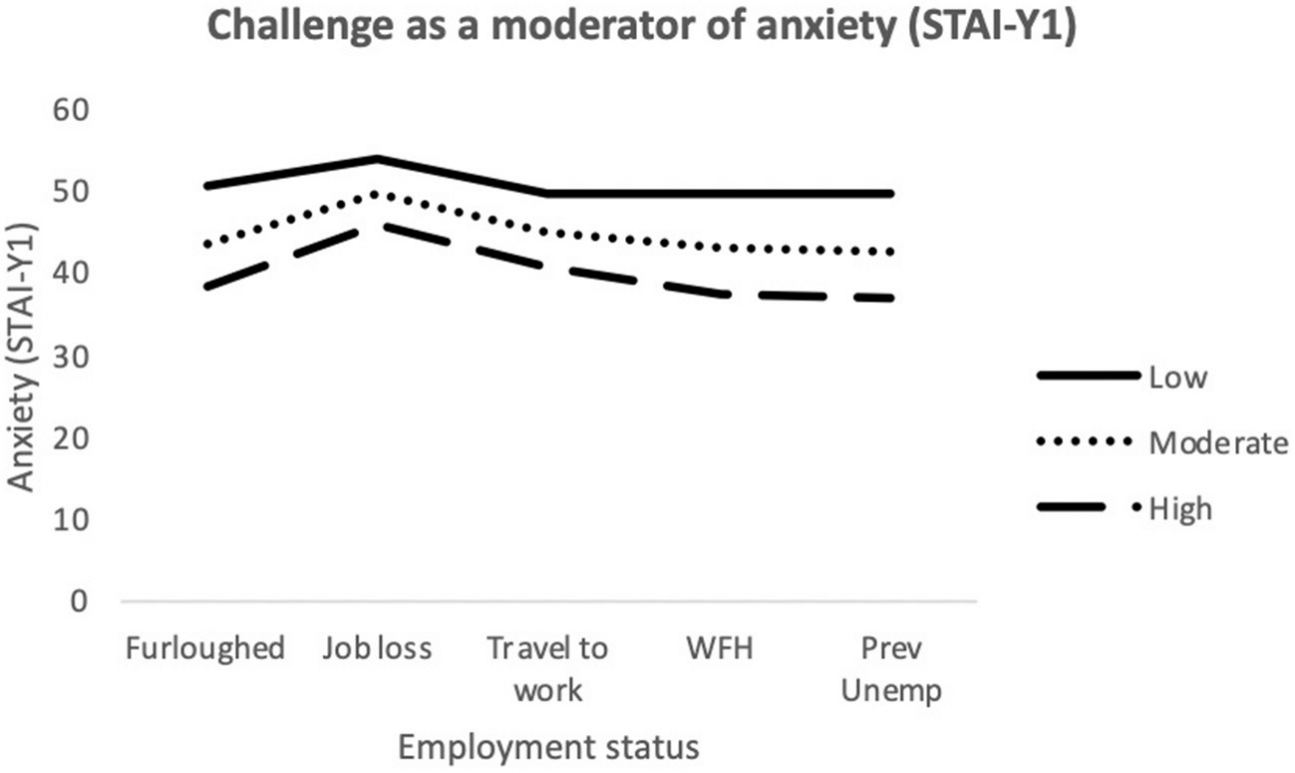
Interaction between job outcome and Challenge for State Anxiety. *WFH*, Employed (working from home); *Prev Unemp*, previously unemployed.

**Figure 2:**
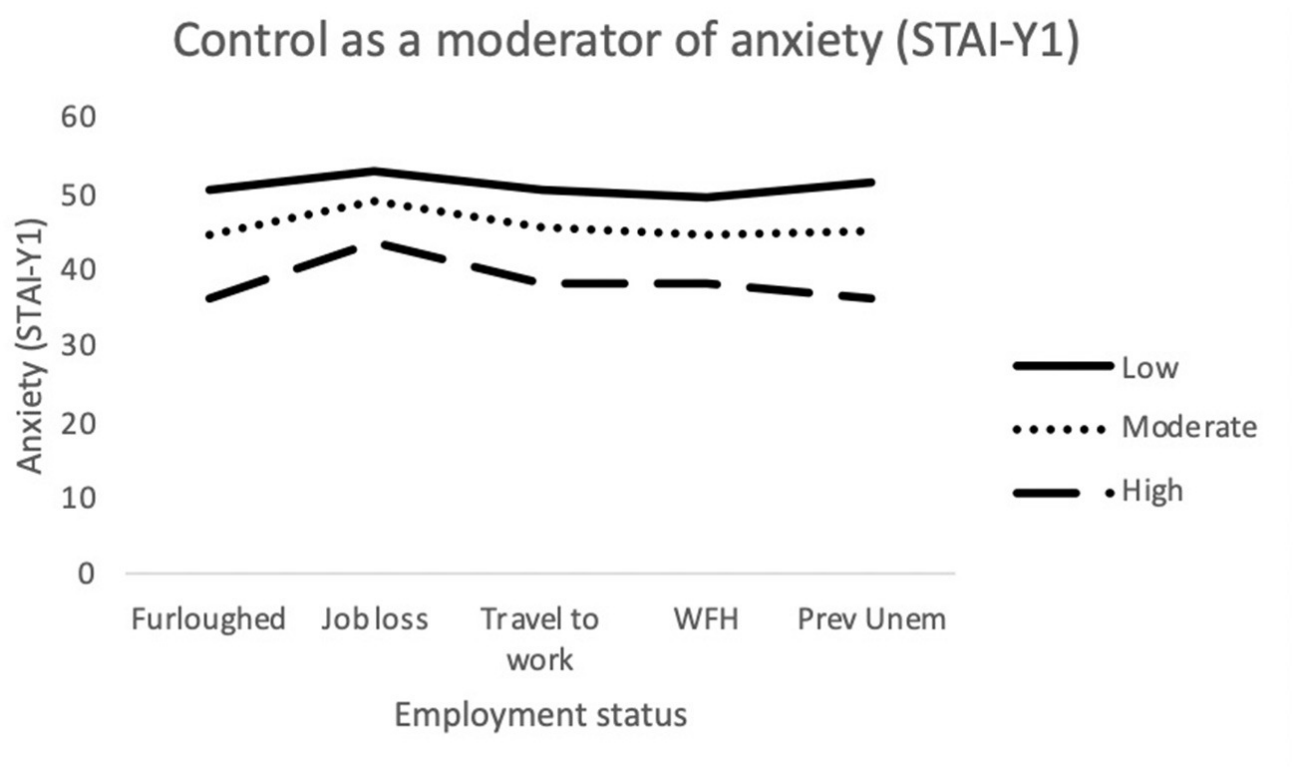
Interaction between job outcome and Control for State Anxiety. WFH, Employed (working from home); Prev Unemp, previously unemployed.

The final model for state anxiety (STAI-Y1) was statistically significant [*F*_(24,676)_ = 22.99; *p* < 0.001] and explained 44.9% of variance. Job loss, Challenge, Control and Confidence made a significant contribution to the model. The interaction terms *working (traveling) x Challenge* and *furloughed x Control* were also significantly associated with state anxiety (see Figures 1, 2, respectively).

**Figure 3:**
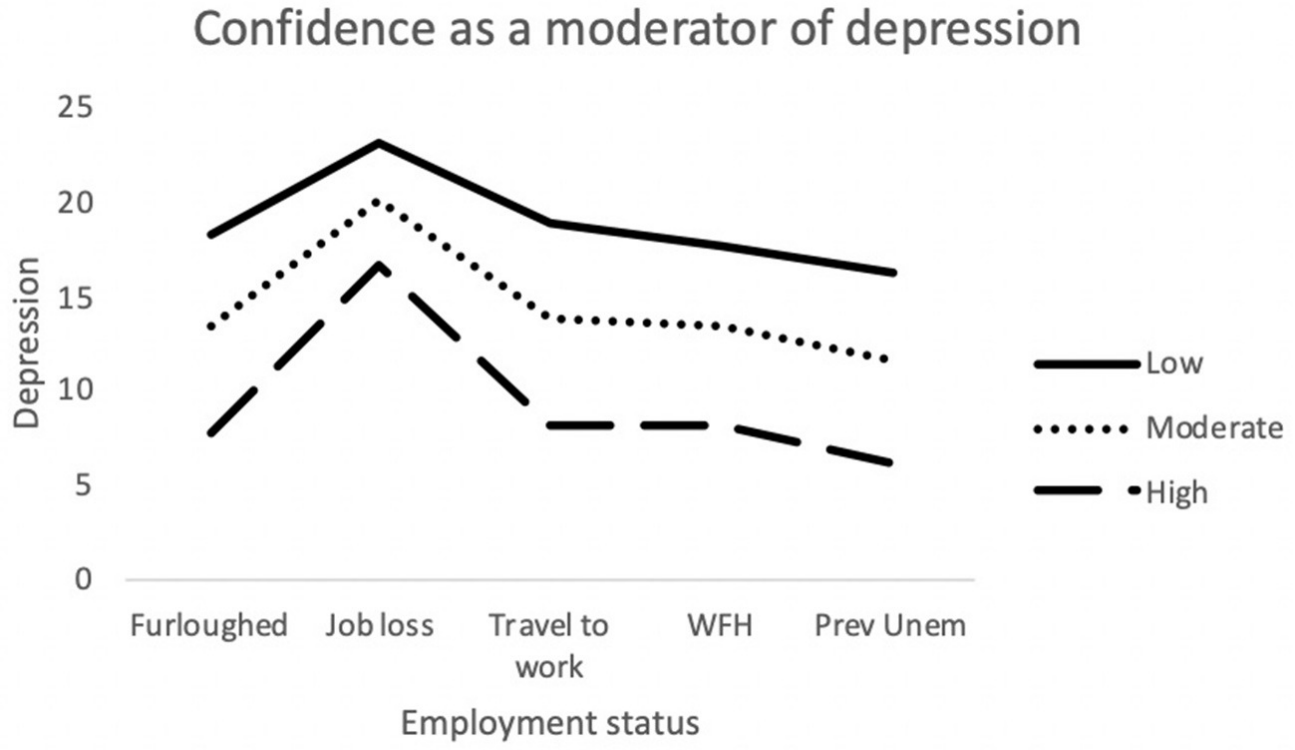
Interaction between job outcome and Confidence for Depression. WFH, Employed (working from home); Prev Unemp, previously unemployed.

The final model for depression was statistically significant [*F*_(24,690)_ = 25.76; *p* < 0.001] and explained 47.3% of variance. Job loss, Commitment and Control made a significant contribution to the model. The interaction term *furloughed x Confidence* was also significantly associated with depression (see Figure 3).

The final model for anxiety (DASS21) was statistically significant [*F*_(24,689)_ = 16.98; *p* < 0.001] and explained 37.2% of variance. Job loss, Commitment and Control made a significant contribution to the model. There were no significant associations between the outcome and the interaction terms.

**Figure 4:**
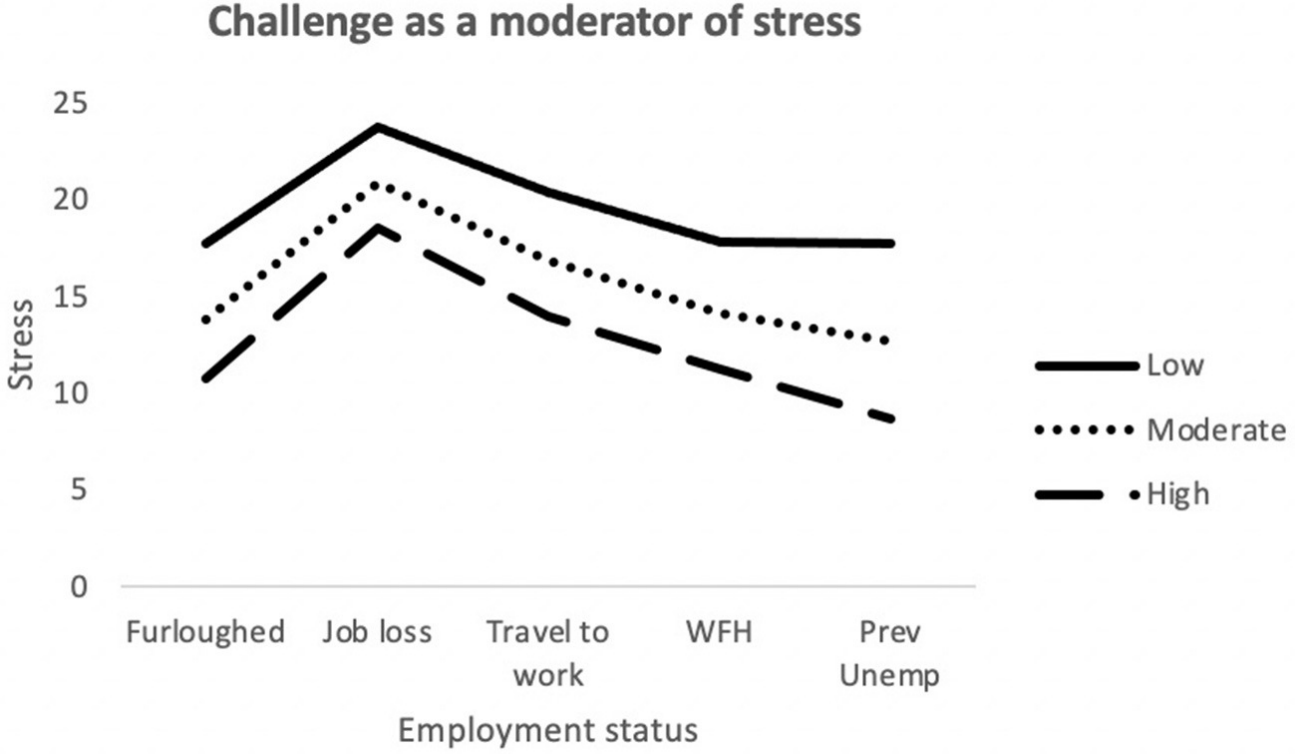
Interaction between job outcome and Challenge for Stress. WFH, Employed (working from home); Prev Unemp, previously unemployed.

**Figure 5:**
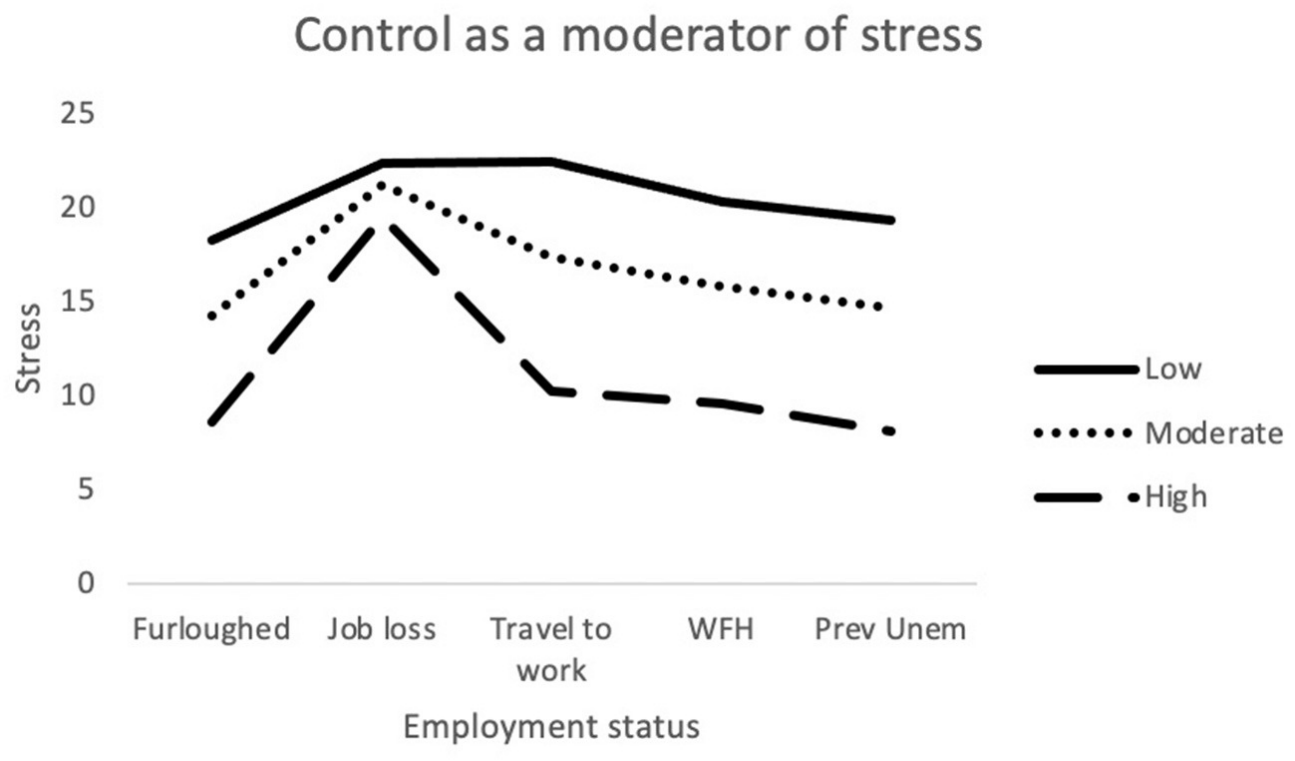
Interaction between job outcome and Control for Stress. WFH, Employed (working from home); Prev Unemp, previously unemployed.

The final model for stress was statistically significant [*F*_(24,689)_ = 19.8; *p* < 0.001] and explained 40.8% of variance. Job loss, Challenge, Commitment and Control made a significant contribution to the model. The interaction terms *job loss x Challenge* and *Job loss x Control were* also significantly associated with stress (see Figures 4, 5).

## Discussion

Individuals who had lost their jobs during the pandemic reported higher levels of depression, anxiety and stress; and lower levels of MT, compared to those who had remained employed. However, across the samples, self-reported symptoms of depression, anxiety and stress were less severe among mentally tough individuals, with MT having a moderating effect on the impact of employment status on mental health. The present study's findings, and their practical implications, are discussed further below.

Participants from Sample B reported higher scores on the DASS21 subscales in comparison to Sample A. One possible explanation for the differences in scores could be the timing of data collection periods, with the majority of participants from Sample B completing the survey at a later period during the pandemic (on May 25th, 2020). Higher death rates, stricter lockdown regulation and greater financial impact on the economy may have led to higher reports of stress, anxiety and depression. However, findings from other COVID-19 studies found that DASS21 scores remained stable throughout the initial lockdown period [see (15)]. The demographic differences between the samples could also explain the differences in scores. Sample A was an exclusively UK cohort whereas Sample B was comprised of participants from a diverse range of countries. Although DASS21 scores appear to be consistent cross-culturally (62), differences in each country's response to controlling the virus may have resulted in changes to the negative affective states of the participants – further evidence on individuals' attitudes to the pandemic would be needed to support this assertion. Other demographic factors that could explain the higher DASS21 scores within Sample B include a greater proportion of men and individuals who had lost their jobs during the pandemic. However, due to the demographic and methodological (data collection platform and time) differences between the two datasets, it is not possible to confidently identify the lead contributor for the disparity in DASS21 scores. More importantly, our discussion of the results focusses on the disparities in negative affective states between the current samples and pre-COVID-19 samples from previous research.

Participants from both samples reported noticeably higher levels of depression, anxiety and stress than participants from previous pre-COVID-19 research. Crawford et al. (63) reported mean scores of 5.14, 3.48, 7.98, and 36.35 for depression, anxiety (DASS21), stress and STAI-Y1, respectively, for Australian adults during 1995–2000^1^. This was proportionately lower than the mean scores of participants from Samples A (10.99, 7.01, 13.32, and 42.7) and B (16.32, 15.2, 17.48, and 44.36). Whilst we cannot definitively confirm the cause for the disparity in scores, it is possible that the distinct differences in affective states could be a result of situational factors brought on by the recent pandemic – however, this ascription should only be taken as speculation due to Crawford's sample not being studied by the present researchers.

### Association Between MT and Negative Affective States

We hypothesized that MT traits would be negatively associated with depression (*H1*), anxiety (*H2*), and stress (*H3*). Partial correlations identified significant negative associations between all MT traits and the depression, anxiety and stress measures, supporting the first three hypotheses. However, after entering the predictors into hierarchical regression models and controlling for trait anxiety, the association between some of the MT traits and the negative affective states were not significant. The reduction in significant associations could be attributed to the high levels of inter-correlation between the MT traits and trait anxiety reducing the additional contribution each predictor made to the model. There were more significant associations between the MT predictors and negative affective states within Sample B, with all four forms of MT being associated with depression, anxiety (DASS21) and stress; and the Control variable being associated with STAI-Y1 as well. The increased level of associations within Sample B could be due to the participants' higher levels of anxiety, stress and depression accentuating the observed relationships between MT and the aforementioned states. Additionally, and unexpectedly, some of the associations between MT and negative affective states appeared to show a positive relationship in the final regression models. This was most notable within Sample B, where the Challenge and Confidence traits had positive standardized coefficients when predicting stress, depression and anxiety (DASS21); and within Sample A, where Commitment had a positive coefficient when predicting STAI-Y1. However, the directions of these associations were not reflected in the initial partial correlations, nor were they present within the moderation analysis of the combined sample, leading the authors to speculate that they may have been the result of a *suppressor effect* from other MT traits.

The relationship between MT and stress can be explained using the *cognitive-transactional stress* theory. According to the model, stress is provoked when the perceived demands of a situation outweigh an individual's ability to cope with the stressor (64). Haghighi and Gerber (38) explained that mentally tough individuals may perceive events as being less stressful due to perceiving their selves as having greater control over the situation, being more capable of staying committed under stress and being better equipped to overcome the issue. Furthermore, Clough et al. (35) defined the Challenge element of MT as the ability to regard problematic events as challenges rather than threats. The same characteristics that allow mentally tough individuals to perceive threatening situations as being less stressful can also reduce the level of anxiety they exhibit. That is, mentally tough individuals with greater confidence in their abilities and perceived control over stressful events are less likely to worry or exhibit fear over them. The relationship between depression and MT was also to be expected, given the clear incompatibility between MT traits and depressive symptoms (i.e., hopelessness, withdrawal and avoidance) (38).

#### Changes in Employment Status During the Pandemic

Results indicated that job/business loss was a significant predictor of anxiety, depression and stress, supporting hypotheses 4, 5, and 6. Our findings align with previous research that had identified a link between job loss and depressive symptoms (65, 66), and also with more recent research showing a relationship between temporary job loss and stress during the COVID-19 pandemic (2). Mimoun and colleagues explained that jobs “provide individuals a sense of confidence, self-esteem, and control” (2, p. 184). Thus, the removal of one's employment is likely to reduce their sense of value and purpose, consequently leading to an increase in depressive symptoms. The effects of recent unemployment with stress and anxiety were also to be expected as the economic hardship brought on by unemployment can often provoke heightened levels of stress and anxiety amongst individuals lacking financial stability (67).

Moderation analyses were used to assess the utility of MT as a protective factor against the adverse effects brought on by recent changes in employment. Multiple significant interactions between MT and employment status were identified when attempting to predict depression, anxiety and stress. For anxiety (STAI-Y1), there were significant interactions between traveling to work and Challenge scores. As illustrated in Figure 1, among participants who were traveling to work during the pandemic, those who possessed low levels of the Challenge trait still exhibited greater levels of anxiety, despite still being in employment. The Challenge characteristic is defined as an individual's tendency to adapt to changing environments and perceive potential threats as opportunities for growth (35). As such, individuals scoring low on this trait may have been less able to overcome the changes in their work environments and more likely to worry about the increased risk of viral contamination. There was also an interaction effect between getting furloughed and Control scores. Furloughed individuals with high Control scores reported less anxiety than those with moderate and low scores. Furloughed individuals with high levels of perceived Control may see temporary unemployment as a more manageable and solvable issue and would be better equipped to manage their emotions whilst awaiting their return to work. For depression, there was a significant interaction between getting furloughed and Confidence. It is possible that individuals with higher levels of confidence would be less likely to interpret temporary unemployment as a reflection of their professional worth and thus, would be less likely to experience depressive symptoms as a result. Finally, for stress, job loss significantly interacted with both Challenge and Control. The perceived stressfulness of an event is influenced by the individual's perceived ability to cope with the new threat (68). Research has shown that high MT is associated with both coping self-efficacy and coping effectiveness (69, 70). Furthermore, individuals who score low on the Challenge scale are less able to adapt to changing environments than individuals with higher scores. As a result, the loss of employment is likely to be handled less effectively by people who are not mentally tough. Individuals with high levels of Control generally reported lower levels of stress in comparison to participants with low or moderate levels. However, Control did not appear to provide much protection against stress for those who had lost their job/business during the pandemic. As Figure 5 illustrates, participants with high levels of Control still reported high levels of stress, similar to the levels reported by participants with lower levels of Control. The findings suggest that whilst the Control element of MT can allow individuals to cope with stress better during the pandemic, the perceived stressfulness of unemployment during the pandemic may outweigh their perceived abilities to deal with situation.

#### Stability of Mental Toughness During the Pandemic

The present findings suggest that MT could be susceptible to environmental influence. Whilst the present study's cross-sectional design cannot prove that changes in employment status will have had a direct impact on MT, comparisons between the employment status groups indicated that those who had lost their jobs/business or become furloughed reported lower levels of MT than those who were working from home. Sudden loss of employment can have a negative impact on an individual's perceived level of control over their life and confidence in their own abilities. Previous research supports the notion of MT being a dynamic trait, however changes in MT have typically been measured in relation to growth over time (59, 71); our findings suggest that MT may also be susceptible to regressing.

Our observations suggest that despite the protective utility for mental health, MT is susceptible to environmental influence. Based on these observations, we argue that attempts to preserve and strengthen public MT should and could be attempted by health organizations. The notion of using MT building strategies to improve the well-being of individuals has been proposed in the past. Gucciardi and Jones (44) proposed using interventions that targeted MT as a way of improving the well-being of vulnerable individuals and Gerber et al. (37) argued that training MT would be particularly useful for supporting the mental health of individuals who may be difficult to reach with more typical health interventions. The potential for improving MT through clinical practice has been evidenced within sport-related contexts. Psychological skills training (PST) interventions [e.g., (72)] have been successful in using routine coaching activities (including goal setting, visualization, relaxation and thought stoppage) to enhance psychological qualities that underpin MT (i.e., hardiness, self-esteem, self-efficacy, dispositional optimism, positive affectivity) (73, 74). PST interventions could therefore provide individuals vulnerable to stress, depression and anxiety during a pandemic– such as recently unemployed individuals– with the necessary psychological prerequisites to maintain emotional resilience. This is because the aforementioned qualities of MT are incompatible with symptoms of stress and depression (e.g., irritability and hopelessness) and as such could mitigate the adverse effects of a pandemic.

## Limitations and Directions for Future Research

The present study is the first to examine the role of MT as a protective factor for mental health during the Covid-19 pandemic. Undoubtably, some limitations exist that require acknowledgment. Firstly, the cross-sectional design of the study meant that we were unable to measure the respondents' MT and state of mental health before or at the early stages of the pandemic. As a result, we cannot reliably determine how much of an impact the pandemic had on MT and mental health. A longitudinal design would have enabled us to assess the utility of MT as a protective factor against the pandemic's adverse effects on mental health more accurately by observing the interactions between MT and time on self-reported stress, anxiety and depression. Similarly, without a longitudinal design, it is difficult to confidently determine the extent to which MT can be influenced by environmental factors such as job loss. Whilst it is impossible to retrospectively assess MT and mental health scores of individuals at the start of the pandemic, new longitudinal research monitoring individuals' MT scores as society continues to adapt to the effects of COVID-19 could provide further insight into the stability of MT during a pandemic. Building on this, future research should examine whether interventions aimed at improving MT could succeed and whether the interventions can lead to reductions in stress, anxiety and depression.

A second data collection (Sample B) was conducted to gather a larger sample for inferential testing and to determine whether the associations between MT and negative affective states could be replicated within a sample that differed geographically. Unfortunately, we were unable to control or match the sample for other potential extraneous variables (i.e., time of data collection, gender and employment distribution). Due to multiple salient differences between the samples, it is not possible to reliably ascribe an explanation for the differences in negative affective state.

Finally, our study measured employment status through five nominal categories (job/business loss during the pandemic, furloughed, traveling to work, working from home and previously unemployed) but failed to distinguish those who were retired or students. It is possible that these individuals may have had a confounding effect on the observed relationship between employment status and mental health. This is possible given that many students have reported experiencing greater psychological impact due to disruption to their educational environments (75). In addition to this, the conditions of temporary unemployment (furloughed) will have differed for each participant. Whilst many furloughed individuals within the UK still received some financial support during the period, this was not the case for many others (2). Thus, we acknowledge that a more precise measure of employment could have provided us with a more complete understanding of the effects of the pandemic on different groups.

## Conclusion

The observed severity of depression, anxiety and stress within our samples highlight the psychological impact of the current climate, however the results also suggest that MT could supress some of these effects. Thus, the practical implications of the present findings highlight the potential for MT-based interventions to be used as a means for boosting individuals' resilience to the adverse mental health effects of the pandemic. Past research has demonstrated that not only can MT-related traits (such as hardiness and positive affectivity) be enhanced through PST, but that such enhancements could help build up resilience to negative emotions within stressful situations. Despite this, our understanding of the mental impact of the pandemic is still at a relatively early stage and further longitudinal research is required to better understand the psychological consequences of COVID-19. A practical step forward from the current research would be to determine whether MT can be improved through short-term interventions and whether such an approach could help improve the emotional resilience of individuals during a pandemic.

## Data Availability Statement

The datasets presented in this study can be found in online repositories. The names of the repository/repositories and accession number(s) can be found at: https://osf.io/zebhj.

## Ethics Statement

This study involving human participants was reviewed and approved by the ethics review panel from the University of Huddersfield. The patients/participants provided their written informed consent to participate in this study.

## Author Contributions

The project was created by DM and PC. DM conducted the data analysis, data collection, and manuscript writeup. ND contributed to the writeup of the introduction and methodology and provided guidance on the research design. AD contributed to the writeup of the results. PC contributed to the writeup of the introduction and design of the study. KP provided guidance on the analytical protocol, study design, and contributed to the writeup of the results. SH contributed to the design of the study and to data collection. DC and CL contributed to data collection and the editing of the manuscript. All authors contributed to the article and approved the submitted version.

## Conflict of Interest

The authors declare that the research was conducted in the absence of any commercial or financial relationships that could be construed as a potential conflict of interest.

## Footnotes

^1^The raw DASS21 scores were multiplied by 2, as instructed by the DASS21 manual [see (49)] to make the scores comparable to the present samples'.
